# 5-Aminosalicylic Acid Alters the Gut Bacterial Microbiota in Patients With Ulcerative Colitis

**DOI:** 10.3389/fmicb.2018.01274

**Published:** 2018-06-13

**Authors:** Jun Xu, Ning Chen, Zhe Wu, Yang Song, Yifan Zhang, Na Wu, Feng Zhang, Xinhua Ren, Yulan Liu

**Affiliations:** ^1^Department of Gastroenterology, Peking University People’s Hospital, Beijing, China; ^2^Clinical Center of Immune-Mediated Digestive Diseases, Peking University People’s Hospital, Beijing, China; ^3^Institute of Clinical Molecular Biology and Central Laboratory, Peking University People’s Hospital, Beijing, China

**Keywords:** ulcerative colitis, 5-aminosalicylic acid, mucosal microbiota, bacterial dysbiosis, bacterial correlation

## Abstract

**Background:** The aim of this study was to clarify the effect of 5-aminosalicylic acid (5-ASA) treatment on gut bacterial microbiota in patients with ulcerative colitis (UC).

**Methods:** A total of 57 UC patients, including 20 untreated and 37 5-ASA-treated, were recruited into an exploration cohort. We endoscopically collected both non-inflamed and inflamed mucosal samples from all patients, and compared the gut bacterial profiles using *16S rDNA* sequencing. Ten untreated UC patients were then treated with 5-ASA and subsequently recruited for an independent validation study to confirm the acquired data.

**Results:** In untreated UC patients, compared with those in non-inflamed mucosae, Firmicutes (such as *Enterococcus*) were decreased and Proteobacteria (e.g., *Escherichia–Shigella*) were increased in the inflamed mucosae. Compared with the inflamed mucosae of untreated UC patients, there was a higher abundance of Firmicutes (e.g., *Enterococcus*) and lower Proteobacteria (*Escherichia–Shigella*) in the inflamed mucosae of 5-ASA treated UC patients. In the validation cohort, after administration of 5-ASA, bacterial alteration was consistent with these data. Furthermore, there was a skewed negative correlation between *Escherichia–Shigella* and bacterial genera of Firmicutes in the inflamed mucosae. 5-ASA treatment decreased the strength of bacterial correlation and weakened the skewed negative correlation pattern.

**Conclusion:** The microbial dysbiosis (mainly characterized by an increased abundance in the *Escherichia–Shigella* genus) and the skewed negative correlation between *Escherichia–Shigella* and bacterial genera of Firmicutes are two characteristics of the inflamed mucosae of UC patients. 5-ASA treatment decreases *Escherichia–Shigella* and weakens the skewed correlations, which may be related to its treatment efficiency.

## Introduction

Ulcerative colitis (UC), a major form of inflammatory bowel disease (IBD), is an idiopathic chronic inflammatory condition affecting the colon and rectum, which influences life quality of patient (Ungaro et al., 2017). Although the pathogenesis of IBD remains unclear, it is accepted that bacterial dysbiosis is an important cause (Sartor and Wu, 2017). Bacterial dysbiosis in IBD is characterized by a reduction in bacterial diversity, a decrease in the Firmicutes phylum (*Faecalibacterium, Blautia*, *Roseburia*, etc.), and an increase in the Proteobacteria phylum (Enterobacteriaceae, including *Escherichia*) (Ott et al., 2004; Liguori et al., 2016; Sokol et al., 2016; Takahashi et al., 2016). Compared with healthy subjects, an increase in fecal Proteobacteria and a decrease in Firmicutes have been observed in IBD patients; additionally, compared with patients in remission, the mucosae of patients in an active stage were colonized with a higher abundance of Proteobacteria and a lower abundance of Firmicutes (Liguori et al., 2016; Sokol et al., 2016). Apart from bacterial imbalance, there are some reports on the intestinal microbiome in IBD patients, including fungal and viral microbiomes (Li et al., 2014; Norman et al., 2015; Liguori et al., 2016; Sokol et al., 2016; Butto and Haller, 2017; Hedin et al., 2017; Pascal et al., 2017).

It is reported that there was relative connection between inflammation and bacterial dysbiosis in IBD pathogenesis. For example, intestinal dysbiotic microbiota triggers a sustained and uninhibited inflammatory response by inducing effective cells, such as type 1, 9, and 17 T helper cells and innate lymphoid cells, to produce pro-inflammatory cytokines like interferon-γ, IL-17 and tumor necrosis factor α (Kamada et al., 2013; Rooks and Garrett, 2016; Belkaid and Harrison, 2017; Ungaro et al., 2017). Adversely, it is also reported that inflammation drove microbial dysbiosis (Zeng et al., 2017). Several studies provide evidence that the inflammatory tissue facilitates a growth advantage for pathogens such as *Citrobacter rodentium* and *Salmonella* (Barman et al., 2008; Kamada et al., 2012; Zeng et al., 2017). Thus, it is plausible for regulating inflammatory status to affect the microbiota. Nevertheless, the effect of regulating inflammation on the bacterial microbiota has been rarely studied in IBD patients.

Currently, 5-aminosalicylic acid (5-ASA), an anti-inflammatory modulator, is the primary therapeutic regimen for controlling inflammation in IBD patients (Laharie, 2017; Ungaro et al., 2017). Andrews et al. (2011) reported that 5-ASA altered fecal bacterial microbiota in patients with irritable bowel syndrome (IBS). In this study, 12 women with diarrhea-predominant IBS received 5-ASA treatment. Data from *16S rRNA* sequencing showed a decrease of bacterial operational taxonomic units (OTUs), and an increase in the abundance of Firmicutes (Andrews et al., 2011). However, the effect of 5-ASA on mucosal bacterial microbiota in UC patients is still unclear. Therefore, we aim to clarify the effect of 5-ASA treatment on the bacterial microbiota in this study to gain insight into the probable causal relationships associated with 5-ASA therapy.

## Materials and Methods

### Study Subjects and Biopsy Collection

This study was approved by the Institutional Medical Ethics Review Board of Peking University People’s Hospital. All UC patients were enrolled from Peking University People’s Hospital from January 2015 to January 2017. The diagnosis of UC was established according to the World Gastroenterology Organization Global Guidelines (Bernstein et al., 2016). Apart from patients who did not undergo mucosal sampling, 57 patients in the activate stage of UC were recruited for an exploration cohort. Two groups, untreated (*n* = 20) and 5-ASA-treated (*n* = 37), were included in this cohort. Only patients following regimens for at least 1 month were classified in the 5-ASA-treated group. Furthermore, 10 UC patients from the 20 untreated patients in the exploration cohort underwent 5-ASA treatment for approximately 6 months and showed a decrease in their Mayo Endoscopic score (described below), but not a clinical complete remission. All of them were recruited into a validation cohort to confirm the data acquired from the exploration cohort (**Table 1** and Supplementary Table S1). All recruited patients were requested to avoid using probiotics and antibiotics for at least 2 weeks before sampling. After that, for each patient, inflamed mucosae and adjacent non-inflamed (2–4 mm^3^ of each mucosa sample) mucosae were obtained endoscopically and stored at -80°C after freezing in liquid nitrogen until DNA was extracted. We defined “non-inflamed” and “inflamed” by using endoscopic observation (Lewis et al., 2008). The mucosa with following characters was considered as “endoscopic inflamed,” including erythema, decreased/absent vascular pattern, friability, erosions, spontaneous bleeding, or ulceration. The mucosa without these symptoms was defined as “endoscopic non-inflamed.”

**Table 1:** Demographic and clinical data of UC patients in the exploration cohort.

Treatment		Untreated	5-ASA treated	*p*-Value
Number		20	37	–
Gender	Male/Female	12/8	9/28	0.032^∗^
Age	Mean ± SD, year	48 ± 14	47 ± 16	0.796
Mayo clinic score	Mean ± SD	2.3 ± 0.6	2.1 ± 0.7	0.516
(Endoscopic)	Normal or inactive (0)	0 (0%)	0 (0%)	–
	Mild (1)	1 (5%)	6 (16%)	–
	Moderate (2)	13 (65%)	20 (54%)	–
	Severe (3)	6 (30%)	11 (30%)	–
Montreal	E1	4 (20%)	10 (27%)	–
classification	E2	7 (35%)	11 (30%)	–
	E3	9 (45%)	16 (43%)	–

*Untreated, untreated UC patients; 5-ASA treated, 5-ASA treated UC patients. ^∗^*p* < 0.05*.

### DNA Extraction

Microbial genomic DNA was extracted from biopsy samples using the QIAamp DNA Stool Mini Kit (Qiagen, Hilden, Germany) according to the manufacturer’s instructions, with minor modifications. Briefly, each biopsy sample was re-suspended in 200 μL phosphate-buffered saline with 80 μL enzyme solution (22.5 mg lysozyme powder [Sigma-Aldrich, United States] and 40 units of mutanolysin dissolved in 80 μL 10 mM Tris-HCl/1 mM ethylenediaminetetraacetic acid [Sigma, United States]). After a 40-min incubation at 37°C, 2 zirconium beads (0.1 mm) were added, and the mixtures were homogenized in a Mini-bead Beater (FastPrep, United States) (Chen et al., 2012). The subsequent genomic DNA-purification steps were performed according to the manufacturer’s instructions.

### *16S rDNA* Amplification and Sequencing

After DNA extraction, bacterial *16S rDNA* was amplified. Briefly, the V3–V4 region of *16S rDNA* was amplified using paired primers (357F: CCTACGGGNBGCASCAG/806R: GACTACNVGGGTATCTAATCC). The *16S rDNA* gene was PCR-amplified using the KAPA HiFi HotStart PCR Kit (Kapa Biosystems, United States) in a 25-μL reaction volume containing 0.5 μL KAPA HiFi HotStart DNA Polymerase, 5 μL GC buffer, 0.5 μL deoxyribonucleoside triphosphates, 0.5 μM of each primer, 2 μL genomic DNA, and 16 μL double-distilled water. The reaction was held at 95°C for 3 min, followed by 25 cycles at 95°C for 1 min, 55°C for 30 s, and 72°C for 30 s, with a final elongation step at 72°C for 5 min in an ABI thermocycler (Applied Biosystems 2720, United States). Each PCR product was purified and amplified again to link with sample-specific barcodes (NEXTflex^TM^ DNA PCR Master Mix, Bioo Scientific, United States). After quantification using an ND-1000 v3.3.0 spectrophotometer (NanoDrop, United States), a paired-end sequencing (2 × 125 bp) was performed on an Illumina HiSeq 2500 sequencer in two lanes at the Center for Molecular Immunology of Chinese Academy of Sciences (Beijing, China).

### *16S rDNA* Sequence Analysis

The Illumina reads were sorted into different samples according to their barcoded index sequences. Fast Length Adjustment of SHort reads (FLASH) software was used to merge paired-end reads from the next-generation sequencing results (Magoc and Salzberg, 2011). Low-quality reads were filtered using the fastq_quality_filter (-p 90 -q 25 -Q33) in FASTX-Toolkit, v.0.0.14 (Caporaso et al., 2010) and chimera reads were removed with USEARCH 64 bit, v8.0.1517 (Edgar et al., 2011). The OTUs were aligned utilizing the UCLUST algorithm with a 97% identity and taxonomically classified using the SILVA database, v128 released on 29/09/2016 (Quast et al., 2013). Alpha and beta diversity were generated with the Quantitative Insights Into Microbial Ecology (QIIME) (Caporaso et al., 2010) pipeline and calculated based on weighted and unweighted Unifrac-distance matrices. The pivotal criterion to select core OTUs was an abundance higher than 10 reads in at least 1 sample. We used the linear discriminant analysis (LDA) effect size (LEfSe) method to identify species with statistically significant differential abundance among groups (Segata et al., 2011). We characterized species alpha diversity in the community by calculating the Shannon and Chao1 diversity indexes. A Venn diagram was drawn for analysis of group-specific bacterial microbiota. In addition, we analyzed bacterial beta diversity using partial-least squares discrimination analysis (PLS-DA) and non-metric multi-dimensional scaling (NMDS) (Caporaso et al., 2010). Additionally, the relative abundance of the various phyla, classes, orders, families, and genera in each sample was computed and compared among all groups.

### Microbial Abundance-UC Severity Correlation in the Exploration Cohort

The Mayo Endoscopic Score (Lewis et al., 2008) was used to estimate the severity of UC patients. Based on the total Mayo Clinic score, UC patients were divided into four groups: inactive (Mayo score = 0, *n* = 0), mild (Mayo score = 1, *n* = 7), moderate (Mayo score = 2, *n* = 33), and severe (Mayo score = 3, *n* = 17). Linear regression was performed to analyze correlations between microbial abundance and UC severity.

### Analysis of the Bacterial Interaction Patterns

To analyze bacterial-interaction patterns associated with different treatment strategies, pairwise bacterial abundance at the genus level was analyzed to determine correlations using Spearman’s method. Correlation coefficients were calculated using the *pandas* software package of Python, v.3.6.0. Gplots and pheatmap packages were launched in R 3.3.2 and Cytoscape 3.4.0, respectively, to visualize the patterns of microbial-interaction networks. Only significant correlations (*p*-value < 0.05 after false-discovery rate correlation) are shown.

### Data Availability Statement

The sequences generated in the present study are available through the NCBI Sequence Read Archive (accession number SRP136321).

### Statistical Analysis

GraphPad Prism, v.6.0c was used for data analysis and graph preparation. All data are expressed as the mean ± standard error of the mean. An analysis of variance (ANOVA) with Fisher’s least significant difference *post hoc* test was used for data analysis. Differences with a *p*-value < 0.05 were considered statistically significant.

## Results

### General Information on Recruited People

Fifty-seven UC patients, including untreated (*n* = 20) and 5-ASA treated (*n* = 37), were recruited for the exploration cohort. There was no significant difference in mean age between the untreated and 5-ASA treated groups (*p* = 0.796). The endoscopic mayo clinic score and Montreal classification of each patient were recorded (**Table 1**). The Mayo Endoscopic score in the untreated and 5-ASA treated groups was not significantly different (2.3 ± 0.6 vs. 2.1 ± 0.7, *p* = 0.516).

Ten UC patients were recruited for an independent validation study; the mean age was 33 ± 14 years, and four out of 10 patients were male. The mean therapy time was 6 months. Six patients were treated with 5-ASA for approximately 6 months, and four patients were treated for approximately 12 months. The total Mayo Endoscopic score of each patient was recorded before and after 5-ASA treatment. After 5-ASA treatment, the total mayo clinic score of UC patients was decreased significantly (9.2 ± 2.5 vs. 5.9 ± 1.8, *p* = 0.002) (**Table 2**).

**Table 2:** Demographic and clinical data of UC patients in the validation cohort.

5-ASA treatment		Before	After	*p*-Value
Numer		10		–
Gender	Male/Female	4/6		–
Age	Mean ± SD, year	33 ± 14		–
Median therapy time	Months	6		–
Mayo clinic	Mean ± SD	9.2 ± 2.5	5.9 ± 1.8	0.002^∗∗^
score (Total)	Normal or inactive (0–2)	0 (0%)	0 (0%)	–
	Mild (3–5)	2 (20%)	4 (40%)	–
	Moderate (6–10)	4 (40%)	6 (60%)	–
	Severe (11–12)	4 (40%)	0 (0%)	–
Montreal	E1	5 (50%)		–
classification	E2	2 (20%)		–
	E3	3 (30%)		–

*Before, before 5-ASA treatment; After, after 5-ASA treatment. ^∗∗^*p* < 0.01*.

### Effect of 5-ASA on Bacterial Diversity in UC Patients

#### Different Traits of the Bacterial Diversity in Untreated and 5-ASA Treated UC Patients of the Exploration Cohort

**FIGURE 1:**
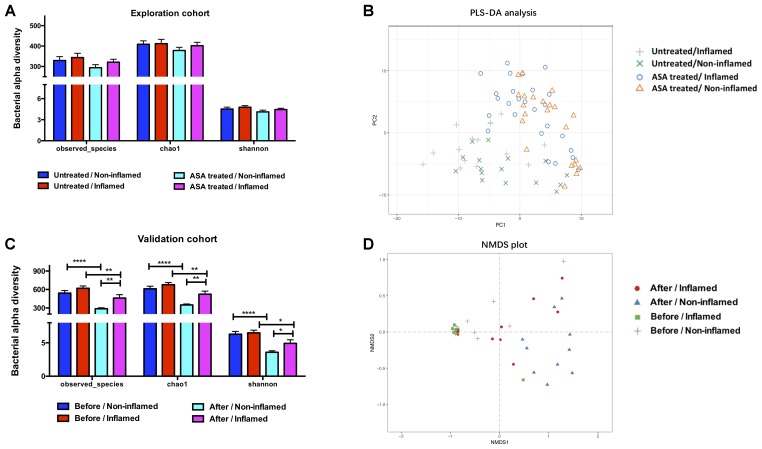
The bacterial diversity in the exploration and validation cohorts. **(A)** The bacterial alpha diversity in the exploration cohort. **(B)** Partial least-squares discrimination analysis (PLS-DA) of the bacterial beta diversity in exploration cohort. **(C)** The bacterial alpha diversity in the validation cohort. **(D)** Non-metric multidimensional scaling (NMDS) analysis of the bacterial beta diversity in the validation cohort. Untreated/Non-inflamed, non-inflamed mucosae of untreated UC patients; Untreated/Inflamed, inflamed mucosae of untreated UC patients; ASA-treated/Non-inflamed, non-inflamed mucosae of 5-ASA treated UC patients; ASA-treated/Inflamed, inflamed mucosae of 5-ASA treated UC patients. Before/Non-inflamed, non-inflamed mucosae before 5-ASA treatment; Before/Inflamed, inflamed mucosae before 5-ASA treatment; After/Non-inflamed, non-inflamed mucosae after 5-ASA treatment; After/Inflamed, inflamed mucosae after 5-ASA treatment.

The bacterial alpha diversity was analyzed using observed species, and Chao1 and Shannon’s indexes, and there was no significant difference between the Untreated/Non-inflamed and Untreated/Inflamed groups (**Figure 1A**). PLS-DA was carried out to estimate bacterial beta diversity. The bacterial microbiota clustered depending on non-inflamed and inflamed mucosae (**Figure 1B**).

We further analyzed the bacterial contents in the ASA-treated/Inflamed group. No significant difference in alpha diversity was found between the Untreated/Inflamed and ASA-treated/Inflamed groups (**Figure 1A**). Analyzing the bacterial beta diversity indicated that 5-ASA treatment drove separate clustering of all samples obtained from inflamed mucosae (**Figure 1B**).

#### 5-ASA Treatment Altered the Bacterial Diversity of UC Patients in the Validation Cohort

Determining the Chao1 and Shannon indexes for the observed species revealed no statistical differences between the groups. Irrespective of whether the mucosae were non-inflamed or inflamed, 5-ASA treatment significantly decreased the bacterial alpha diversity. After 5-ASA treatment, however, higher alpha diversity was observed in After/Inflamed group compared with After/Non-inflamed group (**Figure 1C**).

In place of PLS-DA, NMDS analysis was performed to confirm the effect of 5-ASA treatment on bacterial beta diversity. We observed mucosal-type-dependent and treatment-dependent clustering in all mucosal samples (**Figure 1D**). These data indicated that 5-ASA treatment significantly altered the mucosal bacterial diversity.

### Effect of 5-ASA on the Bacterial Composition in UC Patients

#### Different Traits of the Bacterial Composition in Untreated and 5-ASA Treated UC Patients of Exploration Cohort

**FIGURE 2:**
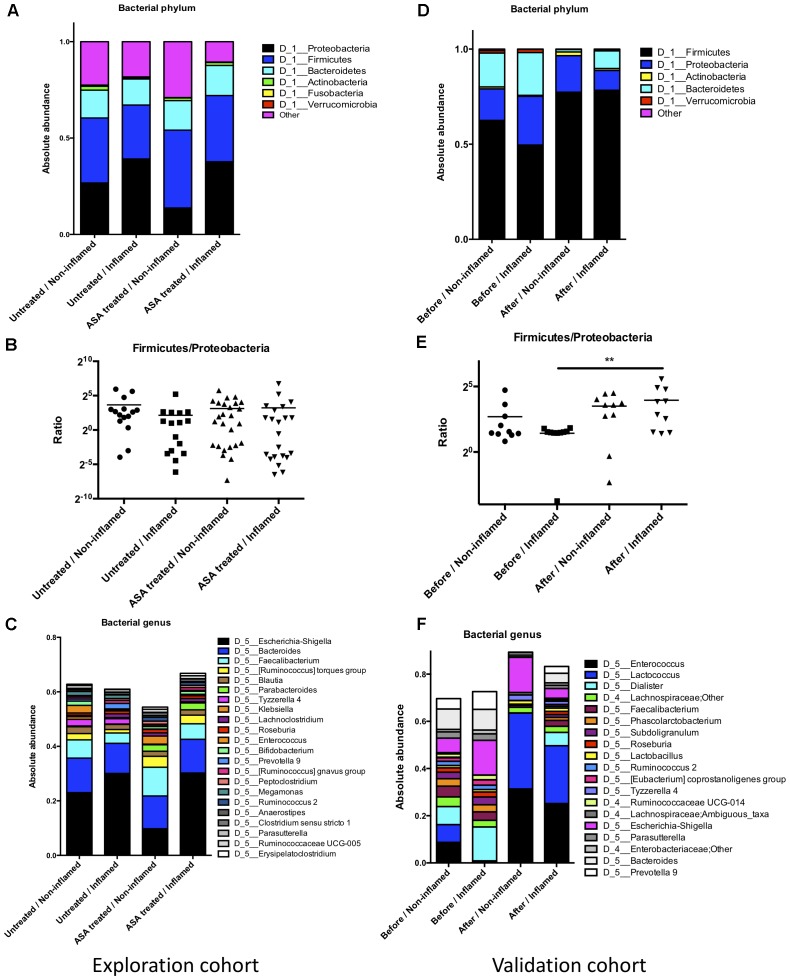
The bacterial composition in the exploration and validation cohorts. **(A)** The bacterial composition at the phylum level in the exploration cohort. **(B)** Firmicutes/Proteobacteria abundance ratio in the exploration cohort. **(C)** The bacterial composition at the genus level in the exploration cohort. **(D)** The bacterial composition at the phylum level in the validation cohort. **(E)** The bacterial composition at the phylum level in the validation cohort, ^∗∗^*p* ≤ 0.01. **(F)** Firmicutes/Proteobacteria abundance ratio in the validation cohort. Untreated/Non-inflamed, non-inflamed mucosae of untreated UC patients; Untreated/Inflamed, inflamed mucosae of untreated UC patients; ASA-treated/Non-inflamed, non-inflamed mucosae of 5-ASA treated UC patients; ASA-treated/Inflamed, inflamed mucosae of 5-ASA treated UC patients. Before/Non-inflamed, non-inflamed mucosae before 5-ASA treatment; Before/Inflamed, inflamed mucosae before 5-ASA treatment; After/Non-inflamed, non-inflamed.

Firmicutes, Bacteroidetes, and Proteobacteria phyla constituted the main part of the bacterial microbiota. These data were consistent with a previous study (Sokol et al., 2016). Of note, there were trends of abundant decrease in Firmicutes and increase in Proteobacteria in the Untreated/Inflamed group compared with the Untreated/Non-inflamed group (**Figures 2A,B**), although these differences were not significant. Additionally, at the genus level, most of the bacterial microbiota were comprised of *Escherichia–Shigella*, *Bacteroides*, and *Faecalibacterium*. Compared with the Untreated/Non-inflamed group, there were decreased trends in abundance of *Enterococcus* and *Faecalibacterium*, and increased trends in the abundance of *Escherichia–Shigella* and *Prevotella_9* in the Untreated/Inflamed group (**Figure 2C**).

Compared with the Untreated/Inflamed group, a lower abundance of Proteobacteria and a higher abundance of Firmicutes were noted in the ASA treated/Inflamed group at the phylum level, but these differences were not significant (**Figures 2A,B**). At the genus level, *Faecalibacterium* and *Bifidobacterium* were more abundant, and *Escherichia–Shigella* and *Prevotella_9* were less abundant in the ASA treated/Inflamed group, compared with the Untreated/Inflamed group (**Figure 2C**).

#### 5-ASA Treatment Altered the Bacterial Composition of UC Patients in the Validation Cohort

We further analyzed the bacterial composition in the validation cohort. At the phylum level, a lower abundance of Firmicutes and a higher abundance of Bacteroidetes and Proteobacteria were observed in the Before/Inflamed group compared with the Before/Non-inflamed group, although these differences were not statistically different. After 5-ASA treatment, the abundance of Firmicutes significantly increased, and the Bacteroidetes abundance significantly decreased in the inflamed mucosae (After/Inflamed vs. Before/Inflamed) (**Figure 2D** and Supplementary Table S2). In addition, the Firmicutes/Proteobacteria abundance ratio significantly increased in the inflamed mucosae (*p* = 0.004) (**Figure 2F**).

At the genus level, 19 bacterial genera were found with an average abundance of over 1%. These genera belonged to three phyla: Firmicutes (14), Proteobacteria (3), and Bacteroidetes (2) (**Figure 2E**). Thirty genera represented over 0.5% of the total and belonged to four phyla: Firmicutes (16), Proteobacteria (9), Bacteroidetes (4), and Verrucomicrobia (1) (Supplementary Table S3).

Before 5-ASA treatment, compared with the non-inflamed group, 4 bacterial genera (*Prevotella_2*, *Prevotella_9*, *Dialister*, and *Klebsiella*) significantly increased in the inflamed group. The abundance of some bacteria changed in the inflamed group without statistical significance. Trends toward alterations in these genera, such as *Subdoligranulum*, *Roseburia*, *Eubacterium coprostanoligenes*, and *Escherichia-Shigella* increased, and *Enterococcus*, *Lactococcus*, and *Faecalibacterium* decreased in inflamed mucosae (Supplementary Table S3).

Compared with the untreated groups, after 5-ASA treatment, the abundance of *Enterococcus* and *Lactococcus* increased significantly in both non-inflamed and inflamed mucosae. However, the abundance of several bacterial genera such as *Bacteroides*, *Prevotella_9*, *Faecalibacterium*, *Phascolarctobacterium*, *Subdoligranulum*, *Roseburia*, *Ruminococcus_2*, *Eubacterium coprostanoligenes*, *Ruminococcaceae UCG-014*, *Ruminococcaceae_UCG_002*, *Dialister*, *Lachnospiraceae_NK4A136_group*, *Parasutterella*, and *Akkermansia* significantly decreased after 5-ASA treatment (Supplementary Table S3). These data suggested that 5-ASA treatment altered the bacterial composition.

Based on these data, we primarily hypothesized that 5-ASA treatment affected the bacterial microbiota. To test this hypothesis, we subsequently performed a further confirmation study in the validation cohort.

### 5-ASA Treatment Altered the Representative Bacteria in the Validation Cohort

LEfSe analysis was performed to identify the differential bacteria composition. The difference in the bacterial microbiota between non-inflamed and inflamed mucosae was also explored using the Mann–Whitney *U* test at different taxon levels, including order, family and genus.

**FIGURE 3:**
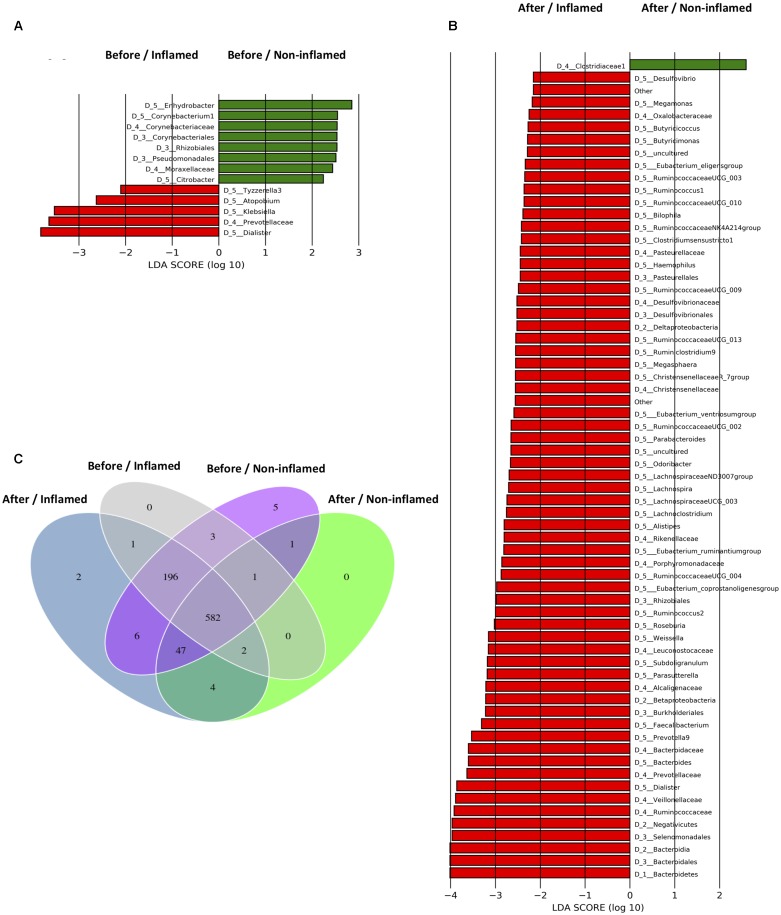
Taxonomic differences of bacterial microbiota and specific operational taxonomic units (OTUs) of samples in the validation cohort. **(A)** Bacterial taxa differentially abundant before 5-aminosalicylic acid (5-ASA) treatment. **(B)** Bacterial taxa differentially abundant after 5-ASA treatment. Differential-abundance microbial cladogram obtained by LEfSe. **(C)** Venn diagram representing the specific and shared bacterial microbiota. Before/Non-inflamed, non-inflamed mucosae before 5-ASA treatment; Before/Inflamed, inflamed mucosae before 5-ASA treatment; After/Non-inflamed, non-inflamed mucosae after 5-ASA treatment; After/Inflamed, inflamed mucosae after 5-ASA treatment.

Before 5-ASA treatment, three representative bacterial genera were identified in the Before/Non-inflamed group and four representative genera were identified in the Before/Inflamed group. *Tyzzerella_3*, *Atopobium*, *Klebsiella*, and *Dialister* were enriched in the Before/Inflamed group (**Figure 3A**). To investigate the effect of 5-ASA treatment on mucosal microbiota, the differentiated taxa in the After/Non-inflamed and After/Inflamed groups were assessed. Forty representative genera were found in the After/Inflamed group, but no representative bacterium was found in the After/Non-inflamed group, at the genus level. Firmicutes members in the Clostridiales order (Christensenellaceae, Lachnospiraceae, and Ruminococcaceae) and Selenomonadales order (Veillonellaceae) were overrepresented in the After/Inflamed group. Among the Firmicutes, the *Roseburia*, *Faecalibacterium*, *Ruminococcaceae UCG_002/003/004/009/010/013*, *Ruminococcus_2*, and *Lachnos piraceaeUCG_003* genera were associated with the After/Inflamed group (**Figure 3B**). These data indicated that Firmicutes were overrepresented in mucosae after 5-ASA treatment, especially in inflamed mucosae.

### Group-Specific Bacterial Microbiota in the Validation Cohort

To further investigate the effects of 5-ASA treatment on the bacterial microbiota, we analyzed group-specific OTUs before and after 5-ASA treatment. The group-specific OTUs were identified by matching data with the SILVA database. We identified 850 OTUs, 582 of which were shared by all four groups (**Figure 3C**).

*Rhodanobacter* (OTU420, Proteobacteria) uniquely existed in non-inflamed mucosae (Before/Non-inflamed and After/Non-inflamed groups), and OTU843 exclusively colonized inflamed mucosae (Before/Inflamed and After/Inflamed groups). Five specific OTUs were found in the Before/Non-inflamed group. Four of them were not identified in the SILVA database, although one OTU was identified in the database (*Anaerosalibacter*, a genus belonging to the Clostridiaceae family). Three OTUs, namely *Phascolarctobacterium* (OTU51, Firmicutes), *Bacteroides* (OTU146, Bacteroidetes), and OTU837, were shared by the Before/Non-inflamed and Before/Inflamed groups. No unique bacteria were detected in the Before/Inflamed and After/Non-inflamed groups. Several new OTUs were discovered after 5-ASA treatment. *Ruminiclostridium* (OTU164, Firmicutes) and OTU805 were found in the After/Inflamed group, and *Clostridium sensu stricto 2* (OTU393, Firmicutes), *Bacillus* (OTU597, Firmicutes), *Coprococcus 2* (OTU624, Firmicutes), and OTU821 were found in both the After/Non-inflamed and After/Inflamed groups (Supplementary Table S4).

These data suggested that more kinds of Firmicutes bacteria and less kinds of Proteobacteria and Bacteroidetes bacteria existed in the intestinal mucosae of UC patients after 5-ASA treatment.

### 5-ASA Treatment Altered the Bacterial Interaction Patterns in UC Patients

**FIGURE 4:**
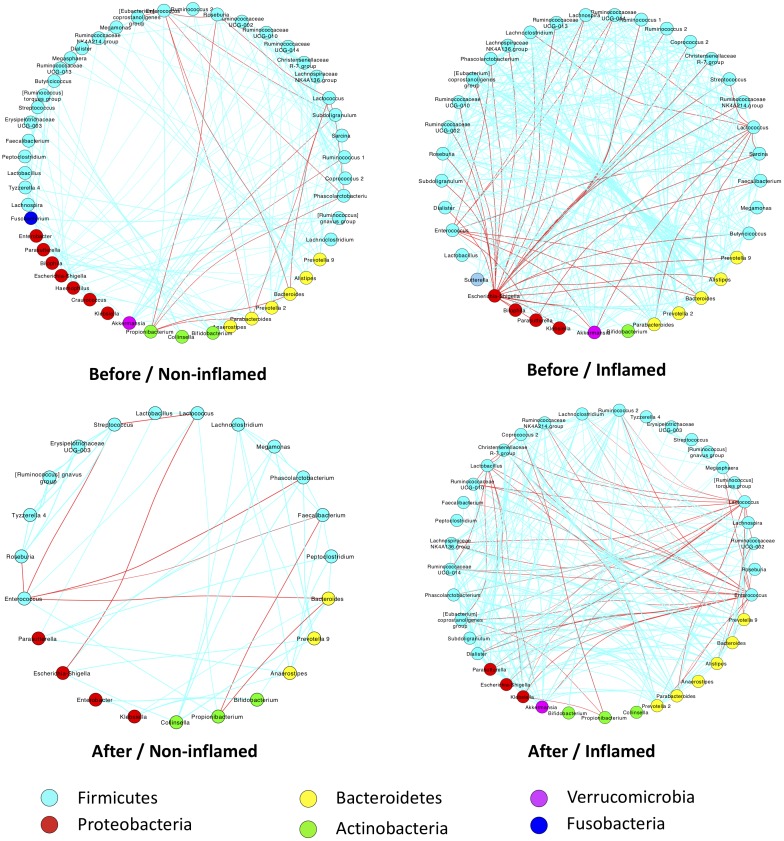
5-ASA treatment altered bacterial-interaction patterns in UC patients of the validation cohort. Bacterial abundances were analyzed using Spearman’s test. Only significant correlations (*p*-value < 0.05) are displayed with an edge. The edge colors indicate positive (green) or negative (red) correlations, which depended on Spearman’s correlation coefficient. The nodes represent microbial genera – the colors of which represent bacterial phyla. Before/Non-inflamed, non-inflamed mucosae before 5-ASA treatment; Before/Inflamed, inflamed mucosae before 5-ASA treatment; After/Non-inflamed, non-inflamed mucosae after 5-ASA treatment; After/Inflamed, inflamed mucosae after 5-ASA treatment.

We further analyzed the effect of 5-ASA treatment on bacterial interaction patterns in non-inflamed and inflamed mucosae. Before 5-ASA treatment, the abundance of *Lactococcus, Enterococcus*, and *Roseburia* (belonging to Firmicutes) correlated negatively with *Bacteroides* and *Propionibacterium* in non-inflamed mucosae. Additionally, a low degree of bacterial correlation was found in the Before/Non-inflamed group. Notably, there was an extensive negative correlation of Escherichia–Shigella with Firmicutes (Ruminococcaceae, *Faecalibacteria, Streptococcus, Lachnoclostridium,* etc.) and Bacteroidetes (*Prevotella, Alistipes, Bacteroides,* and *Parabacteroides*) in the Before/Inflamed group. In addition, *Bacteroides* and *Parabacteroides* (belonging to the Bacteroidetes phylum) negatively correlated with *Lactococcus* and *Enterococcus.* These data indicated that increased Bacteroidetes and Proteobacteria abundance negatively correlated with the Firmicutes abundance in the Before/Inflamed group. These skewed bacterial correlations tended to be related to inflammatory responses in inflamed mucosae (**Figure 4**).

After 5-ASA treatment, a few bacterial correlations were observed in the After/Non-inflamed group. Compared with the Before/Inflamed group, fewer bacterial correlations were observed in the After/Inflamed group. *Escherichia–Shigella* did not extensively correlate with Firmicutes. However, the abundance of *Lactococcus* and *Enterococcus* increased and negatively correlated with Bacteroidetes (including *Prevotella* and *Parabacteroides*) and Proteobacteria (including Escherichia–Shigella, *Klebsiella* and *Parasutterella*). These data suggested that 5-ASA treatment alleviated the skewed negative correlation between *Escherichia–Shigella* and Firmicutes. In addition, increased Firmicutes exerted a suppressive effect on Bacteroidetes and Proteobacteria (**Figure 4**).

### Relationship Between 5-ASA Efficiency and the Bacterial Microbiota

#### The Correlation Between UC Severity and Bacterial Abundance in the Exploration Cohort

We assumed that, irrespective of 5-ASA treatment, the abundance of some bacteria correlated with UC severity. To test this hypothesis, linear-regression analysis was performed to analyze the correlation between bacterial abundance and UC severity in the exploration cohort.

**FIGURE 5:**
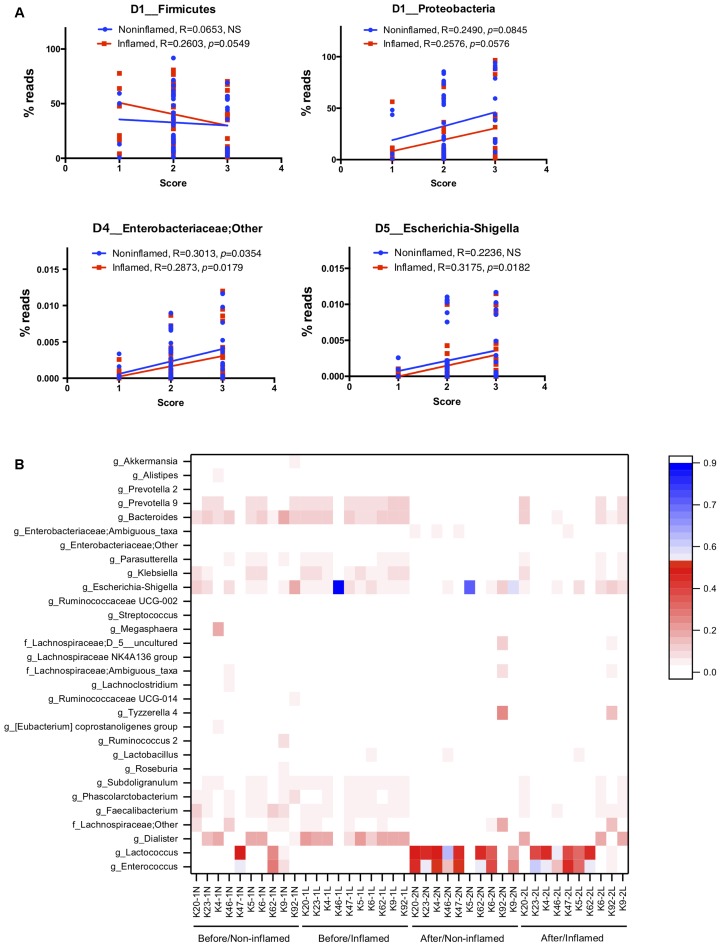
The relationship between 5-ASA treatment efficiency and bacterial microbiota. **(A)** The linear-regression analysis of the correlation between IBD severity and bacterial abundance at the phylum and genus levels in the exploration cohort. **(B)** Clustering heatmap of the bacterial abundance at the genus level in the validation cohort. Apart from unidentified OTUs, 50 bacterial genera of the highest abundance are displayed in this heatmap. Before/Non-inflamed (-1N), non-inflamed mucosae before 5-ASA treatment; Before/Inflamed (-1L), inflamed mucosae before 5-ASA treatment; After/Non-inflamed (-2N), non-inflamed mucosae after 5-ASA treatment; After/Inflamed (-2L), inflamed mucosae after 5-ASA treatment.

We found that bacterial abundance in non-inflamed mucosae poorly correlated with UC severity. In inflamed mucosae, the abundance of Firmicutes (*R* = 0.2603, *p* = 0.0549) negatively correlated with UC severity, and Proteobacteria (*R* = 0.2576, *p* = 0.0576) positively correlated with UC severity at the phylum level. Analysis at the genus level showed that the abundance of *Faecalibacterium* (*R* = 0.2873, *p* = 0.0334), *Roseburia* (*R* = 0.3416, *p* = 0.0107), and *Bifidobacterium* (*R* = 0.3536, *p* = 0.0081) negatively correlated with UC severity, while Enterobacteriaceae (*R* = 0.2873, *p* = 0.0179) and *Escherichia–Shigella* (*R* = 0.3175, *p* = 0.0182) positively correlated with UC severity (**Figure 5A** and Supplementary Table S5). These data indicated that the bacterial abundance might be a reliable factor for evaluating UC severity.

#### Bacterial Abundance Reflected the Efficiency of 5-ASA Treatment

To investigate the relationship between 5-ASA treatment efficiency and bacterial microbiota, we clustered samples by analyzing bacterial abundance at the genus level (**Figure 5B**). We found obvious clustering depending on 5-ASA treatment. Before 5-ASA treatment, bacteria such as *Escherichia–Shigella*, *Dialister*, *Bacteroides*, *Prevotella_9*, and *Klebsiella* colonized in the non-inflamed and inflamed mucosae with high abundance. The abundance of these bacteria decreased after 5-ASA treatment, and increase in *Enterococcus*, *Lactococcus*, and *Lactobacillus* was detected. Additionally, compared with the untreated group, several bacteria such as *Faecalibacterium*, *Subdoligranulum*, *Parasutterella*, *Roseburia*, *Ruminococcus_2*, *Lachnoclostridium*, *Prevotella_2*, *Akkermansia*, *Phascolarctobacterium*, and *Ruminococcaceae UCG-014* decreased after 5-ASA treatment (**Figure 5B**).

In our exploration study, we found that UC severity positively correlated with the abundance of *Escherichia–Shigella* in inflamed mucosae. In the validation study, although the difference was not significant, we still found a decreased abundance of *Escherichia–Shigella* and other genera belonging to Enterobacteriaceae after 5-ASA treatment (Supplementary Table S5). These data indicated that bacterial abundance (e.g., *Escherichia–Shigella*) might reflect the efficiency of 5-ASA treatment.

## Discussion

Compared with fecal microbiota (passersby), mucosal microbiota act as residents that can continuously activate the host immune system and induce chronic gut inflammation (Tang et al., 2015). To investigate the characteristics of the gut microbiota of UC patients undergoing 5-ASA treatment, we analyzed mucosal samples. Additionally, because of ethical limitations, we did not compare the mucosal bacterial microbiota of UC patients to healthy controls. Previous studies have reported microbial differences between non-inflamed and inflamed mucosae, and between remission and inflamed mucosae (Li et al., 2014; Liguori et al., 2016). Therefore, we compared the bacterial microbiota in inflamed and non-inflamed mucosae.

Gut microbiota is a complex biosystem affected by several factors. It has been reported that the location of the mucosal sampling sites influenced the microbial composition (Aguirre de Carcer et al., 2011; Gu et al., 2013; Costa et al., 2015; He et al., 2018). According to the Montreal classification, we enrolled approximately the same percentage of patients with each type to verify this effect. Furthermore, a previous study also reported a sex-based effect on mucosa-associated bacteria along the human colon (Aguirre de Carcer et al., 2011). We found that there was a significant difference in the sex percentage in our exploration cohort. To avoid the sex-based bias in our study, we further confirmed the results obtained from the exploration cohort within our validation cohort.

Bacterial dysbiosis is related to IBD development (Wlodarska et al., 2015; Imhann et al., 2016). In IBD patients, the bacterial diversity and the percentage of the Firmicutes phylum decreased and that of the Proteobacteria phylum increased (Ott et al., 2004; Liguori et al., 2016; Sokol et al., 2016; Takahashi et al., 2016). Our data were in agreement with these previous studies. Recently, some IgA-coated bacteria have been shown as dominant pathogens in IBD pathogenesis (Okai et al., 2017). *Escherichia* (belonging to the Proteobacteria phylum) is a genus of IgA-coated bacteria (Viladomiu et al., 2017). In addition, *Escherichia coli* (in particular, the AIEC pathotype) has been implicated in IBD pathogenesis (Nguyen et al., 2014). It has been reported that chronic infection with *Escherichia* induced cell cycle disorder, DNA damage, and inflammation. These processes play pivotal roles in IBD development (Khan et al., 2017; Viladomiu et al., 2017). Apart from *Escherichia*, a high abundance of Bacteroidetes was discovered in the inflamed mucosa of untreated UC patients, especially in the validation cohort. This finding suggests that the pathogenesis is related to Bacteroidetes species, such as *Bacteroides*, *Prevotella_2*, *Prevotella_9*, and *Parabacteroides*. *Bacteroides fragilis* has been reported to produce a toxin termed *B. fragilis* toxin that induced IBD and even colorectal cancer (Sears, 2009). *Prevotella falsenii* and *Parabacteroides distasonis*, two peptidoglycan recognition protein-regulated gut microbes, were also reported as aggravating colitis (Dziarski et al., 2016). In our exploration study, we also found that UC severity correlated with the Proteobacteria abundance, especially Enterobacteriaceae and *Escherichia–Shigella* in inflamed mucosa. We also assessed the bacterial microbiota in UC patients after 5-ASA treatment in our validation cohort. We found that it reduced the abundance of bacteria associated with inflammation after 5-ASA treatment, such as *Escherichia–Shigella*, *Bacteroides*, *Prevotella_9*, *Prevotella_2*, and *Klebsiella*. These data indicate that 5-ASA treatment might affect inflammation-associated bacteria colonizing the gut mucosa of UC patients.

Short-chain fatty acids (SCFAs), especially butyrate, provide up to 60% of the energy needed to colonic epithelium and gut immune cells, and play beneficial roles in anti-inflammation, anti-carcinogenesis, mucosal protection, and healing (Cook and Sellin, 1998; Cani et al., 2013; van der Beek et al., 2017). SCFAs levels are reduced in IBD patients, and this reduction is associated with a decrease in SCFAs-producing bacteria (Machiels et al., 2014). In Firmicutes, SCFA-producing bacteria include *Blautia*, *Roseburia*, *Ruminococcus*, *Clostridium*, *Faecalibacterium*, etc. In the exploration study, we found that the abundance of *Roseburia*, *Ruminococcus*, *Clostridium*, *Faecalibacterium*, and *Dorea* decreased in the inflamed mucosae of untreated UC patients. These data were in accord with those of previous studies (Machiels et al., 2014; Lopez-Siles et al., 2015, 2016). Additionally, we also showed that UC severity negatively correlated with bacteria such as *Faecalibacterium*, *Roseburia*, and *Bifidobacterium* in our exploration cohort. Although we could not perform a statistical analysis with our validation cohort because of the small sample size, these data were consistent with previous reports (Ott et al., 2004; Morgan et al., 2012; Sokol et al., 2016; Takahashi et al., 2016), and they validated the methods used in this study. Notably, increased colonization of Firmicutes, such as *Ruminiclostridium*, *Clostridium sensu stricto 2*, *Coprococcus 2*, and *Bacillus,* was found after 5-ASA treatment. The former three genera belong to the Clostridiales order and have been associated with SCFAs production (Spalinger et al., 2015). *Bacillus clausii,* a probiotic *Bacillus* spp., is utilized to treat small intestinal bacterial overgrowth (Gabrielli et al., 2009). These data suggest that the gut mucosa developed to a suitable state for colonization of some beneficial Firmicutes after 5-ASA treatment.

Sokol et al. (2017) reported a skewed microbial interaction pattern in IBD patients. He found that the concomitant analysis of microbiota showed a dense and homogenous correlation network in healthy subjects, but an unbalanced network in IBD patients (Sokol et al., 2017). In our validation cohort, the skewed negative correlation between *Escherichia–Shigella* and bacterial genera of Firmicutes was also found in the inflamed mucosae of UC patients before 5-ASA treatment. Many of the involved Firmicutes bacteria were SCFA-producing bacteria. It has been reported that the translocation of *E. coli* across epithelia was reduced by SCFAs, especially butyrate (Lewis et al., 2010; Byndloss et al., 2017). We inferred that the decrease in anti-inflammatory SCFA-producing bacteria, such as some Firmicutes bacteria, and the increase of pro-inflammatory *Escherichia–Shigella* represent two fundamental traits in the mucosal inflammation of UC patients. Thus, the skewed negative correlation between *Escherichia–Shigella* and bacterial genera of Firmicutes in inflamed mucosae may play a key role in gut inflammation in UC patients. After 5-ASA treatment, we found that the skewed interaction disappeared, which partly indicated a therapeutic effect of 5-ASA.

It is widely accepted that there is an interaction between host immunity and microbiota. The gut microbiota plays an instrumental role in the development and education of the host immune system early in life (Gensollen et al., 2016; Gomez de Aguero et al., 2016). Through its symbiotic relationship with immune cells, colonizing microbiota can stimulate host immunity to prevent pathogen invasion (Walker, 2014). The interaction between the immune system and microbiota is essential for the immune defense system of the host in a healthy state (Belkaid and Harrison, 2017). These reports highlighted a microbiota–immunity interaction. Nevertheless, interruption of host immunity–microbiota interactions plays a pivotal role in triggering inflammation in IBD, which is mediated by the host immune system (Du et al., 2015; Wlodarska et al., 2015; Kim et al., 2017; Kramer and Genco, 2017; Pickard et al., 2017); however, we are not clear as to whether inflammatory status affects gut microbiota. We observed in our study that there were parallel changes between the decrease in the Mayo Endoscopic score and bacterial alteration (e.g., decreased *Escherichia–Shigella*) of UC patients after 5-ASA treatment in our validation cohort. A previous study also indicated that the proposed anti-inflammatory and bacteriological effects of 5-ASA were well aligned with factors implicated in IBS pathogenesis (Andrews et al., 2011). We did not use sulfasalazine (SASP) in our research as its sulfonamide group might directly exert an antimicrobial effect. Unlike SASP, 5-ASA exerts its anti-inflammatory effect mainly by inhibiting TNF-α-regulated IκB degradation and NF-κB activation (Yan and Polk, 1999). Thus, we inferred that 5-ASA treatment might alter bacterial microbiota through regulating inflammatory status, although this needs further investigation.

In this study, we showed the microbial dysbiosis (mainly characterized by an abundant increase of *Escherichia–Shigella*) and the skewed negative correlation between *Escherichia–Shigella* and bacterial genera of Firmicutes in the inflamed mucosa of UC patients. In addition, we found that 5-ASA treatment altered the diversity, composition, and bacterial interaction patterns in mucosal samples of UC patients.

## Ethics Statement

This study was carried out in accordance with the *World Medical Association’s Declaration of Helsinki*. The protocol was approved by the *Institutional Medical Ethics Review Board of Peking University People’s Hospital* (Document No. *2016PHB024-01*). All subjects gave *written informed consent* in accordance with the Declaration of Helsinki.

## Author Contributions

YL and JX designed the study. JX and NC performed acquisition of clinical data. JX, ZW, YS, NW, FZ, and XR performed analysis and interpretation of data. JX and YZ drew the figures. JX and YL wrote the manuscript. NC, ZW, and YS revised the manuscript for important intellectual content. NC and YL supervised the study.

## Conflict of Interest Statement

The authors declare that the research was conducted in the absence of any commercial or financial relationships that could be construed as a potential conflict of interest.
