# Supplementary material for: 5-Aminosalicylic Acid Alters the Gut Bacterial Microbiota in Patients With Ulcerative Colitis
Source: Front Microbiol. 2018 Jun 13;9:1274. doi: 10.3389/fmicb.2018.01274 (PMC6008376; doi:10.3389/fmicb.2018.01274)
Supplement: Supplementary file 1 [file Table_1.DOCX]

Supplementary Material

5-aminosalicylic acid alters the gut bacterial microbiota in patients with ulcerative colitis

Xu Jun^1,2,4^, Chen Ning^1,2,4^, Wu Zhe^1,2^, Song Yang^1,2^, Zhang Yifan^1,2^, Wu Na^3^, Zhang Feng^1,2^, Ren Xinhua^1,2^, Liu Yulan^1,2,*^

*** Correspondence:** Liu Yulan: liuyulan@pkuph.edu.cn

**Table S1. Groups of mucosal samples in the validation cohort.** All mucosal samples were divided into four groups depending on mucosal type (noninflamed and inflamed) and treatment (Before and After). Before/Non-inflamed, non-inflamed mucosae before 5-ASA treatment; Before/Inflamed, inflamed mucosae before 5-ASA treatment; After/Non-inflamed, non-inflamed mucosae after 5-ASA treatment; After/Inflamed, inflamed mucosae after 5-ASA treatment.

| **Sample ID** | **Group** | **Sample ID** | **Group** | **Sample ID** | **Group** | **Sample ID** | **Group** |
| --- | --- | --- | --- | --- | --- | --- | --- |
| K92-1N | Before/Non-inflamed | K92-1L | Before/Inflamed | K92-2N | After/Non-inflamed | K92-2L | After/Inflamed |
| K62-1N | Before/Non-inflamed | K62-1L | Before/Inflamed | K62-2N | After/Non-inflamed | K62-2L | After/Inflamed |
| K20-1N | Before/Non-inflamed | K20-1L | Before/Inflamed | K20-2N | After/Non-inflamed | K20-2L | After/Inflamed |
| K4-1N | Before/Non-inflamed | K4-1L | Before/Inflamed | K4-2N | After/Non-inflamed | K4-2L | After/Inflamed |
| K6-1N | Before/Non-inflamed | K6-1L | Before/Inflamed | K6-2N | After/Non-inflamed | K6-2L | After/Inflamed |
| K5-1N | Before/Non-inflamed | K5-1L | Before/Inflamed | K5-2N | After/Non-inflamed | K5-2L | After/Inflamed |
| K46-1N | Before/Non-inflamed | K46-1L | Before/Inflamed | K46-2N | After/Non-inflamed | K46-2L | After/Inflamed |
| K23-1N | Before/Non-inflamed | K23-1L | Before/Inflamed | K23-2N | After/Non-inflamed | K23-2L | After/Inflamed |
| K9-1N | Before/Non-inflamed | K9-1L | Before/Inflamed | K9-2N | After/Non-inflamed | K9-2L | After/Inflamed |
| K47-1N | Before/Non-inflamed | K47-1L | Before/Inflamed | K47-2N | After/Non-inflamed | K47-2L | After/Inflamed |

**Table S2. Bacterial relative abundance at phylum level in the validation cohort.** Before/Non-inflamed, non-inflamed mucosae before 5-ASA treatment; Before/Inflamed, inflamed mucosae before 5-ASA treatment; After/Non-inflamed, non-inflamed mucosae after 5-ASA treatment; After/Inflamed, inflamed mucosae after 5-ASA treatment. ^*^*p*≤0.05, ^**^*p*≤0.01, ^***^*p*≤0.001, ^****^*p*<0.0001.

| Phylum | Before/Non-inflamed | Before/Inflamed | After/Non-inflamed | After/Inflamed | Before/Non-inflamed vs. Before/Inflamed | Before/Non-inflamed vs. After/Non-inflamed | Before/Inflamed vs. After/Inflamed | After/Non-inflamed vs. After/Inflamed |
| --- | --- | --- | --- | --- | --- | --- | --- | --- |
| Firmicutes | 0.6248±0.0472 | 0.4953±0.0476 | 0.7731±0.0833 | 0.7835±0.0577 | 0.14 | 0.093 | 0.002^**^ | 0.904 |
| Bacteroidetes | 0.1781±0.0287 | 0.2247±0.0241 | 0.0130±0.0033 | 0.0929±0.0341 | 0.203 | <0.0001^****^ | 0.001^***^ | 0.033^*^ |
| Actinobacteria | 0.0104±0.0026 | 0.0048±0.0011 | 0.0196±0.0061 | 0.0103±0.0028 | 0.292 | 0.087 | 0.302 | 0.083 |
| Proteobacteria | 0.1659±0.0247 | 0.2568±0.0737 | 0.1922±0.0815 | 0.1041±0.2271 | 0.271 | 0.748 | 0.068 | 0.285 |
| Fusobacteria | 0.0060±0.0054 | 0.0004±0.0001 | 0.0013±0.0005 | 0.0007±0.0002 | 0.160 | 0.234 | 0.949 | 0.872 |

**Table S3. Bacterial relative abundance at genus level in the validation cohort.** Thirty genera in this table are with average abundance of more than 0.5%. Before/Non-inflamed, non-inflamed mucosae before 5-ASA treatment; Before/Inflamed, inflamed mucosae before 5-ASA treatment; After/Non-inflamed, non-inflamed mucosae after 5-ASA treatment; After/Inflamed, inflamed mucosae after 5-ASA treatment. ^*^*p*≤0.05, ^**^*p*≤0.01, ^***^*p*≤0.001, ^****^*p*<0.0001.

| Phylum | Genus | Before/Non-inflamed | Before/Inflamed | After/Non-inflamed | After/Inflamed | Before/Non-inflamed vs. Before/Inflamed | Before/Non-inflamed vs. After/Non-inflamed | Before/Inflamed vs. After/Inflamed | After/Non-inflamed vs. After/Inflamed |
| --- | --- | --- | --- | --- | --- | --- | --- | --- | --- |
| Bacteroidetes | *Bacteroides* | 0.0865±0.0127 | 0.0876±0.0096 | 0.0098±0.0029 | 0.0406±0.0116 | 0.937 | <0.0001^****^ | 0.002^**^ | 0.035^*^ |
| Bacteroidetes | *Prevotella_9* | 0.0440±0.0121 | 0.0756±0.0093 | 0.0023±0.0020 | 0.0290±0.0138 | 0.038^*^ | 0.007^**^ | 0.003^**^ | 0.076 |
| Bacteroidetes | *Alistipes* | 0.0144±0.0046 | 0.0136±0.0017 | 0.0002±0.0000 | 0.0051±0.0019 | 0.843 | 0.001^***^ | 0.027^*^ | 0.201 |
| Bacteroidetes | *Prevotella_2* | 0.0103±0.00305 | 0.0183±0.0018 | 0.0000±0.00001 | 0.0064±0.0031 | 0.023^*^ | 0.004^**^ | 0.001^***^ | 0.067 |
| Firmicutes | *Faecalibacterium* | 0.0457±0.01182 | 0.0358±0.0040 | 0.0087±0.00297 | 0.0252±0.0058 | 0.328 | 0.001^***^ | 0.295 | 0.106 |
| Firmicutes | *Phascolarctobacterium* | 0.0315±0.00742 | 0.0302±0.0038 | 0.0012±0.00049 | 0.0128±0.0046 | 0.844 | <0.0001^****^ | 0.015^*^ | 0.094 |
| Firmicutes | *Subdoligranulum* | 0.0275±0.00608 | 0.0325±0.0040 | 0.0004±0.00010 | 0.0120±0.0045 | 0.41 | <0.0001^****^ | 0.002^**^ | 0.064 |
| Firmicutes | *Megasphaera* | 0.0193±0.01648 | 0.0034±0.0004 | 0.0000±0.00002 | 0.0026±0.0007 | 0.181 | 0.107 | 0.944 | 0.831 |
| Firmicutes | *Roseburia* | 0.0188±0.00284 | 0.0216±0.0025 | 0.0032±0.00202 | 0.0134±0.0037 | 0.485 | <0.0001^****^ | 0.048^*^ | 0.016^*^ |
| Firmicutes | *Ruminococcus_2* | 0.0178±0.00629 | 0.0184±0.0023 | 0.0004±0.00012 | 0.0079±0.0028 | 0.904 | 0.002^**^ | 0.048^*^ | 0.153 |
| Firmicutes | *[Eubacterium] coprostanoligenes group* | 0.0150±0.00378 | 0.0226±0.0025 | 0.0002±0.00007 | 0.0074±0.0031 | 0.061 | 0.001^***^ | <0.0001^****^ | 0.076 |
| Firmicutes | *Lachnoclostridium* | 0.0147±0.00355 | 0.0114±0.0011 | 0.0029±0.00134 | 0.0066±0.0017 | 0.29 | <0.0001^****^ | 0.127 | 0.24 |
| Firmicutes | *Ruminococcaceae_UCG_014* | 0.0137±0.00485 | 0.0198±0.0023 | 0.0001±0.00002 | 0.0060±0.0029 | 0.165 | 0.003^**^ | 0.003^**^ | 0.177 |
| Firmicutes | *Lactobacillus* | 0.0097±0.00255 | 0.0103±0.0043 | 0.0151±0.00301 | 0.0139±0.0032 | 0.91 | 0.268 | 0.442 | 0.816 |
| Firmicutes | *Lachnospiraceae_NK4A136_group* | 0.0081±0.00192 | 0.0109±0.0010 | 0.0005±0.00011 | 0.0050±0.0015 | 0.144 | <0.0001^****^ | 0.003^**^ | 0.024^*^ |
| Firmicutes | *Streptococcus* | 0.0077±0.00267 | 0.0044±0.0004 | 0.0038±0.00223 | 0.0051±0.0014 | 0.237 | 0.161 | 0.803 | 0.636 |
| Firmicutes | *Ruminococcaceae_UCG_002* | 0.0076±0.00189 | 0.0100±0.0016 | 0.0002±0.00007 | 0.0034±0.0010 | 0.202 | <0.0001^****^ | 0.001^***^ | 0.101 |
| Firmicutes | *f_Lachnospiraceae_Ambiguous_taxa* | 0.0054±0.00408 | 0.0002±0.0000 | 0.0103±0.00813 | 0.0062±0.0052 | 0.489 | 0.512 | 0.429 | 0.578 |
| Firmicutes | *f_Lachnospiraceae_g_uncultured* | 0.0038±0.00264 | 0.0012±0.0000 | 0.0103±0.01009 | 0.0070±0.0059 | 0.761 | 0.448 | 0.495 | 0.703 |
| Firmicutes | *Tyzzerella_4* | 0.0026±0.00231 | 0.0002±0.0000 | 0.0232±0.02208 | 0.0142±0.0137 | 0.896 | 0.273 | 0.453 | 0.63 |
| Firmicutes | *Enterococcus* | 0.0872±0.05122 | 0.0045±0.0014 | 0.3133±0.06173 | 0.1641±0.0328 | 0.29 | 0.006^**^ | 0.003^**^ | 0.04^*^ |
| Firmicutes | *Dialister* | 0.0766±0.02408 | 0.1437±0.0193 | 0.0006±0.00013 | 0.0564±0.0261 | 0.025^*^ | 0.012^*^ | 0.004^**^ | 0.059 |
| Firmicutes | *Lactococcus* | 0.0751±0.04565 | 0.0041±0.0017 | 0.3218±0.06070 | 0.2452±0.0630 | 0.316 | 0.001^***^ | 0.001^***^ | 0.28 |
| Proteobacteria | *Escherichia-Shigella* | 0.0616±0.01531 | 0.1471±0.0854 | 0.14990±0.0844 | 0.0414±0.0106 | 0.326 | 0.316 | 0.227 | 0.219 |
| Proteobacteria | *f_Lachnospiraceae_g_Other* | 0.0408±0.00895 | 0.0283±0.0032 | 0.0238±0.01546 | 0.0257±0.0113 | 0.417 | 0.271 | 0.865 | 0.9 |
| Proteobacteria | *Klebsiella* | 0.0291±0.00924 | 0.0506±0.0067 | 0.0022±0.00116 | 0.0196±0.0089 | 0.044^*^ | 0.013^*^ | 0.005^**^ | 0.1 |
| Proteobacteria | *Parasutterella* | 0.0267±0.00649 | 0.0274±0.0035 | 0.0020±0.00105 | 0.0134±0.0048 | 0.912 | <0.0001^****^ | 0.032^*^ | 0.079 |
| Proteobacteria | *f_Enterobacteriaceae_Other* | 0.0102±0.00259 | 0.0163±0.0022 | 0.0078±0.00189 | 0.0105±0.0024 | 0.069 | 0.456 | 0.083 | 0.407 |
| Proteobacteria | *f_Enterobacteriaceae_Ambiguous_taxa* | 0.0062±0.00327 | 0.0006±0.0001 | 0.0231±0.00605 | 0.0104±0.0034 | 0.311 | 0.004^**^ | 0.078 | 0.025^*^ |
| Verrucomicrobia | *Akkermansia* | 0.0126±0.00498 | 0.0167±0.0021 | 0.0001±0.00002 | 0.0067±0.0029 | 0.353 | 0.007^**^ | 0.028^*^ | 0.141 |

**Table S4. Group-specific OTUs in the validation cohort.** Based on Venn diagram, group-specific OTUs are identified by matching data with SILVA database (<https://www.arb-silva.de/search/>). The unmatched OTUs are displayed with a dash. Before/Non-inflamed, non-inflamed mucosae before 5-ASA treatment; Before/Inflamed, inflamed mucosae before 5-ASA treatment; After/Non-inflamed, non-inflamed mucosae after 5-ASA treatment; After/Inflamed, inflamed mucosae after 5-ASA treatment.

| Group | OUT code | ID in SILVA database | Phylum | Genus | Species |
| --- | --- | --- | --- | --- | --- |
| Before/Non-inflamed AND after/non-inflamed | OTU420 | GU134907.1.1468 | Proteobacteria | *Rhodanobacter* | *Bacterium enrichment culture clone SBIa2* |
| Before/Inflamed AND after/inflamed | OTU843 | New.CleanUp.Reference OTU24405 | — | — | *—* |
| Before/Non-inflamed | OTU697 | CCEZ01000065.95.1603 | Firmicutes | *Anaerosalibacter* | *Anaerosalibacter sp. ND1* |
|  | OTU796 | New.CleanUp.Reference OTU9096 | — | — | — |
|  | OTU801 | New.CleanUp.Reference OTU10091 | — | — | — |
|  | OTU842 | New.CleanUp.Reference OTU24357 | — | — | — |
|  | OTU848 | New.CleanUp.Reference OTU25742 | — | — | — |
| Before/Non-inflamed and Before/Inflamed | OTU51 | EF404034.1.1522 | Firmicutes | *Phascolarctobacterium* | *uncultured bacterium* |
|  | OTU146 | HQ789895.1.1430 | Bacteroidetes | *Bacteroides* | *uncultured organism* |
|  | OTU837 | New.CleanUp.Reference OTU22077 | — | — | — |
| After/Inflamed | OTU164 | AB934798.1.1350 | Firmicutes | *Ruminiclostridium* | *uncultured bacterium* |
|  | OTU805 | New.CleanUp.Reference OTU10770 | — | *—* | *—* |
| After/Non-inflamed AND after/inflamed | OTU393 | JX048101.1.1406 | Firmicutes | *Clostridium sensu stricto 2* | *bacterium NLAE-zl-G77* |
|  | OTU597 | FJ984530.1.1482 | Firmicutes | *Bacillus* | *Bacillus horikoshii* |
|  | OTU624 | DQ799915.1.1395 | Firmicutes | *Coprococcus 2* | *uncultured bacterium* |
|  | OTU821 | New.CleanUp.Reference OTU15440 | — | *—* | *—* |

**Table S5. Linear regression analysis of correlation between bacterial abundance and UC severity in the exploration cohort.** Bacterial abundance at phylum and genus level, and the endoscopic mayo scores of UC patients were used for analysis. Only significant correlations are displayed in this table. ^*^*p*<0.05, ^**^*p*<0.01.

| Phylum | Genus | Correlation | Non-inflamed | | Inflamed | |
| --- | --- | --- | --- | --- | --- | --- |
|  |  |  | **R square** | **P value** | **R square** | **P value** |
| Firmicutes | ***—*** | Negative | 0.004266 | 0.6557 | 0.06776 | 0.0549 |
|  | *Faecalibacterium* | Negative | 0.02357 | 0.2923 | 0.08258 | 0.0334^*^ |
|  | *Roseburia* | Negative | 0.08375 | 0.0437^*^ | 0.1167 | 0.0107^*^ |
| Proteobacteria | *—* | Positive | 0.06198 | 0.0845 | 0.06638 | 0.0576 |
|  | *Enterobacteriaceae;g_Other* | Positive | 0.09081 | 0.0354^*^ | 0.1013 | 0.0179^*^ |
|  | *Escherichia-Shigella* | Positive | 0.05001 | 0.1224 | 0.1008 | 0.0182^*^ |
| Actinobacteria | *—* | Negative | 0.01427 | 0.4135 | 0.1554 | 0.0029^**^ |
|  | *Bifidobacterium* | Negative | 0.01396 | 0.4187 | 0.1250 | 0.0081^**^ |
